# Influence of Industrialization and Environmental Protection on Environmental Pollution: A Case Study of Taihu Lake, China

**DOI:** 10.3390/ijerph15122628

**Published:** 2018-11-23

**Authors:** Yan Li, Shenglu Zhou, Zhenyi Jia, Liang Ge, Liping Mei, Xueyan Sui, Xiaorui Wang, Baojie Li, Junxiao Wang, Shaohua Wu

**Affiliations:** 1 School of Geography and Ocean Science, Nanjing University, 163 Xianlin Road, Nanjing 210023, China; dg1627013@smail.nju.edu.cn (Y.L.); zhenyijay@smail.nju.edu.cn (Z.J.); mg1727004@smail.nju.edu.cn (L.G.); baojieli@smail.nju.edu.cn (B.L.); dz1427034@smail.nju.edu.cn (J.W.); wsh@nju.edu.cn (S.W.); 2 Key Laboratory of Coastal Zone Exploitation and Protection, Ministry of Land and Resources, Nanjing 210008, China; 3 School of Chemistry and Chemical Engineering, Nanjing University, 163 Xianlin Road, Nanjing 210023, China; dg1624048@smail.nju.edu.cn; 4 Jiangsu Land Consolidation and Rehabilitation Center, Nanjing 210023, China; xueyan322@sina.com (X.S.); njuwxr@163.com (X.W.)

**Keywords:** environmental protection, heavy metals, industrialization, neural network, PAHs

## Abstract

In order to quantitatively study the effect of environmental protection in China since the twenty-first century and the environmental pollution projected for the next ten years (under the model of extensive economic development), this paper establishes a Bayesian regulation back propagation neural network (BRBPNN) to analyze the typical pollutants (i.e., cadmium (Cd) and benzopyrene (BaP)) for Taihu Lake, a typical Chinese freshwater lake. For the periods 1950–2003 and 1950–2015, the neural network model estimated the BaP concentration for the database with Nash-Sutcliffe model efficiency (NS) = 0.99 and 0.99 and root-mean-square error (RMSE) = 3.1 and 9.3 for the total database and the Cd concentration for the database with NS = 0.93 and 0.98 and RMSE = 45.4 and 65.7 for the total database, respectively. In the model of extensive economic development, the concentration of pollutants in the sediments of Taihu reached the maximum value at the end of the twentieth century and early twenty-first century, and there was an inflection point. After the early twenty-first century, the concentration of pollutants was controlled under various environmental policies and measures. In 2015, the environmental protection ratio of Cd and BaP reached 52% and 89%, respectively. Without environmental protection measures, the concentrations of Cd and BaP obtained from the neural network model is projected to reach 2015.5 μg kg^−1^ and 407.8 ng g^−1^, respectively, in 2030. Based on the results of this study, the Chinese government will need to invest more money and energy to clean up the environment.

## 1. Introduction

After the Chinese economic reform, the Chinese industry has developed rapidly and the Chinese population has continued to grow [1,2,3]. The economy has been developing in an extensive mode, which incurs higher costs and increase the waste of raw materials [4]. Accompanying the decline in environmental quality in China, the concentration of pollutants in the environment is rapidly increasing. Pollutants, such as polycyclic aromatic hydrocarbons, which are represented by benzopyrene (BaP) [5,6,7,8,9,10,11] and the heavy metal contaminant cadmium (Cd) [12,13,14], pose serious threats to our environment and life due to their strong toxicity and carcinogenicity. In the late twentieth century and early twenty-first century, both the government and people in general began to realize the importance of the environment; as such, environmental protection was strengthened and a series of legal measures for environmental protection were enacted [15,16], as shown in Table 1.

Pollutants enter aquatic systems via surface runoff and the fluvial river networks of watersheds or via atmospheric deposition, are adsorbed by suspended matter (mostly on clays and silts), and then accumulated in sediments. Lake sediments are an important sink for contaminants, and they can reflect the quality of aquatic systems and provide long-term records of past environmental conditions [17]. Stratigraphic analyses of pollutants in lacustrine sediments can be used to reconstruct temporal variations in the input of pollutants to lake ecosystems [17,18,19]. The Yangtze River Delta region is China’s most important economic area. It is an important intersection zone between the Yangtze River economic belt and the featured area of implementation for the “One Belt and One Road” policy. It has a pivotal strategic position in the overall situation of China’s national modernization and the all-round opening up pattern. Taihu Lake is an important water supply source in the Yangtze River Delta region. The quality of Taihu Lake water directly affects the industrial development and the health and wellbeing of people in the region. Artificial neural networks (ANNs) have been used to study pollutants [20,21,22,23,24]. ANNs have the ability to detect complex nonlinear relationships between independent and dependent variables, such as the range of the choices of structures of interconnections among components [25]. ANNs have complex formulas in order to represent the relationships between input values and output values [26] and can be used similarly to a regression formula [25].

The Chinese government has implemented a lot of environmental protection measures to control pollution (Table 1), but it is still unknown whether these environmental protection measures have played a role, and if so, to what extent. This paper uses Taihu Lake, a typical Chinese freshwater lake, as an example, taking the end of the twentieth century and early twenty-first century as the time node, and uses an artificial neural network to (1) explore the historical changes of the concentration of pollutants in the sediment, (2) quantitatively study the historical effects of environmental protection measures, and (3) predict the pollutant concentration changes in sediments in the next ten years.

## 2. Materials and Methods

### 2.1. Field Sampling

**Figure 1:**
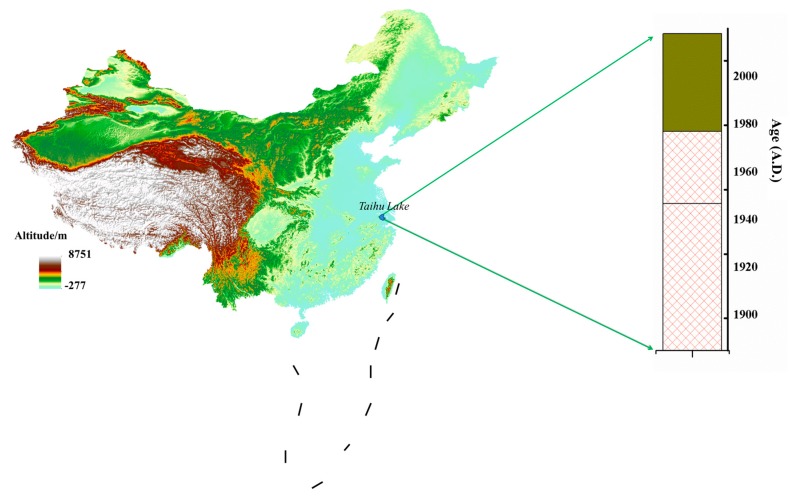
A sedimentary column from Taihu Lake, a typical Chinese freshwater lake

Taihu Lake is the third largest freshwater lake in China and is located in the south of Jiangsu Province, Figure 1. The lake area is 2338 km^2^. It is one of the largest comprehensive industrial bases in China. It plays an important role in the regional social and economic development. A sediment column was sampled from the lake [27] (31.057° N, 120.056° E); it was 45 cm long and was separated at intervals of 1 cm. The column sections were then ground (agate mortar) and passed through a 100-mesh sieve (nylon sieve; less than 150 mm). The samples were stored at −4 °C until they were analyzed.

### 2.2. Sediment Database

The data (1949–2015) on gross industrial output value, energy consumption, and population were obtained from the National Bureau of Statistics (http://www.stats.gov.cn/). The year was calculated by testing the activity of ^210^Pb and ^137^Cs via the use of a high-purity germanium detector (EC & GORTEC, San Louis, CA, USA), digital spectrometer, and multichannel analysis system [27]. The content of Cd was determined by inductively coupled plasma mass spectrometry (ICP-MS, PerkinElmer SCIEX, Elan 9000) [27] (the recovery, detection limit, and operating parameters of ICP-MS are provided in the Supplementary Material, Tables S1 and S2).

The determination of BaP concentration proceeded as follows:

First, we accurately weighed a 5 g soil sample with a scale (accurate to 0.001 g), mixed it with 5 g of anhydrous sodium sulfate, wrapped it with a filter cartridge, and placed it into the suction filter of the cable extractor. In a flat-bottomed flask of the cable extractor, 100 mL of a mixture of methylene chloride and n-hexane (*v*/*v* = 1:1) and 2 g of activated copper pieces were sequentially added, and continuous cable extraction was performed for 24 h. After the extract was concentrated to approximately 2 mL on a rotary evaporator, 10 mL of n-hexane was added, and the mixture was further concentrated to 1 to 2 mL to convert the solvent. Second, the concentrated extract was added to a previously prepared silica gel column for separation and purification. The silica gel column was 25 cm × 1 cm ID, and the bottom was equipped with glass wool and glass columns with Teflon pistons. After the concentrate was applied to the column, it was rinsed with 15 mL of n-hexane and 50 mL of a mixture of dichloromethane and n-hexane (*v*/*v* = 2:3), respectively. The former 15 mL of n-hexane was only used for the rinsing of n-alkanes without collection and disposal. After the collected solution was changed to solvent with n-hexane, it was blown with high-purity nitrogen to 1 mL, placed in a Gas Chromatography (GC) bottle, and tested on the machine. Blanks and matrix blanks were analyzed every six samples during the sample analyses. Duplicates were also run every 12 samples, and the samples were reanalyzed if the difference exceeded ±15% (the recovery and detection limit are provided in the Supplementary Material, Table S1).

### 2.3. Database Analysis

To determine the correlation and significance between variables and contaminants in the database, a correlation analysis of the Pearson correlation coefficient (R) was used. In addition, a univariate nonlinear and linear regression analysis was conducted on the gross industrial output value, total population, and total energy consumption. The results of the analysis were used to determine the input variables when building the neural network.

### 2.4. Artificial Neural Network 

**Figure 2:**
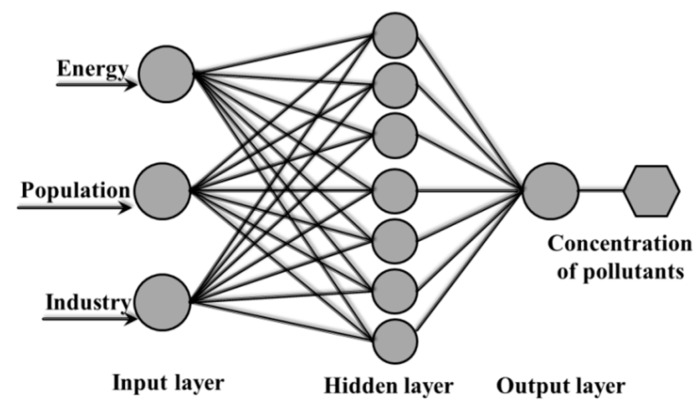
The architecture of the neural networks in this study.

The Back Propagation (BP) network is usually composed of an input layer, a hidden layer, and an output layer [28,29,30]. Each layer is composed of several neurons, and the different levels of neurons are all connected to each other. Through the analysis of the BP network learning process, we can know that the network learning termination condition is to reach the preset training error or training times. If the training error set is too small or the number of trainings is too large, an overfitting is likely to occur, affecting the accuracy of the results. To solve this problem, we used the Bayesian regularization method to train the BP network. We built a three-layer neural network model in this study, as shown in Figure 2. Three factors were selected as the input layer variables. A hidden layer was used, and the concentration of pollutants was the output variable. All the computations were performed using MATLAB (The MathWorks Inc., Natick, MA, USA). The selected transfer function was a hyperbolic tangent sigmoid, and the performance function was the mean square error (MSE). Detailed steps are provided in the Supplementary Material.

### 2.5. Evaluation Criteria

Three different criteria were used to evaluate the performance of the developed BRBPNN model: the root-mean-square error (RMSE), the Nash-Sutcliffe model efficiency (NS), and the coefficient of determination (R^2^) [31,32,33]. The RMSE represents the error associated with the model and is calculated as follows:

(1) $$\mathrm{RMSE}\;=\;\sqrt{\sum_{i=1}^{N}\frac{{\left(E_{i}-M_{i}\right)}^{2}}{N}}\;$$

where *E_i_* and *M_i_* represent the estimated value and the measured values of the variables, respectively, and the *N* represents the number of observations.

The NS is calculated as follows:

(2) $$\mathrm{NS}\;=\;1-\frac{\sum {\left(E_{i}-M_{i}\right)}^{2}}{\sum {\left(E_{i}-\bar{M}\right)}^{2}}\;$$

where $\bar{M}$ is the mean of the measured values.

The coefficient of determination (R^2^) is calculated as follows:

(3) $$R^{2}={\left[\frac{N\sum_{i}^{N}M_{i}E_{i}-(\sum_{i}^{N}M_{i})(\sum_{i}^{N}E_{i})}{\sqrt{\left[N\sum_{i}^{N}M_{i}^{2}-{\left(N\sum_{i}^{N}M_{i}\right)}^{2}\right]}\times \left[N\sum_{i}^{N}E_{i}{}^{2}-{\left(\sum_{i}^{N}E_{i}\right)}^{2}\right]}\right]}^{2}\;$$

## 3. Results

### 3.1. Analysis of Variables and Contaminants

**Figure 3:**
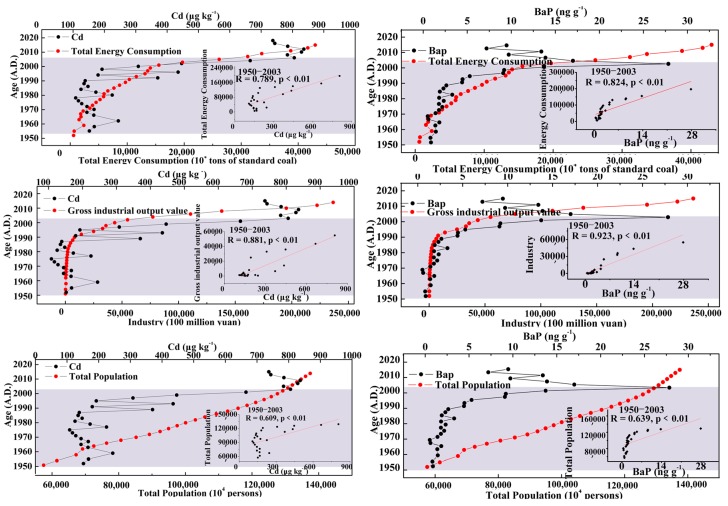
Analysis of correlation and significance between pollutants (benzopyrene (Cd), benzopyrene (BaP)) and Gross Domestic Product (GDP), energy consumption and total population.

To analyze the relationship between the variables and pollutants, the correlation and significance of pollutants Cd and BaP with the industrial output value, energy consumption, and total population were analyzed. The results are shown in Figure 3. The time period under consideration can be roughly divided into two stages: before the 2000s and after the 2000s. Before the 2000s, the concentrations of Cd and BaP have a positive correlation with industrial output, energy consumption, and total population; that is, the concentration of pollutants increases with the growth of industrial output, energy consumption, and total population, which is in accordance with the results of previous studies [17,34,35,36,37,38,39,40,41]. The correlation coefficient of Cd with the industrial output value, energy consumption, and total population is 0.881, 0.789, and 0.609, respectively; the correlation coefficient of BaP with the industrial output value, energy consumption, and total population is 0.923, 0.825, and 0.639, respectively, and all show strong significance (*p* < 0.01). After the 2000s, the concentrations of Cd and BaP no longer showed a positive correlation with industrial output, energy consumption, and total population, but there was a negative correlation to some extent; that is, the concentration of pollutants decreased with the growth of industrial output value, energy consumption, and total population, which coincided with previous results [15,16,27].

### 3.2. BRBPNN Model Training and Test

The BRBPNN model developed for the prediction of pollutant concentrations contains seven neurons and three input variables: the industrial output value, energy consumption, and total population. In practical applications, to improve the speed and sensitivity of training, the value of input data is generally between −1 and 1. This was accomplished using the following formula [42]:

(4) $$\;xf=\frac{\left(xf_{max}-xf_{min}\right)}{\left(x_{max}-x_{min}\right)}\left(x-x_{min}\right)+xf_{min}\;$$

where *xf* is the normalized input variable, *xf_max_* and *xf_min_* are the maximum and minimum value for *xf* equal to −1 and 1, respectively, *x* is the input variable, and *x_max_* and *x_min_* are the maximum and minimum value for the input variable, respectively.

**Figure 4:**
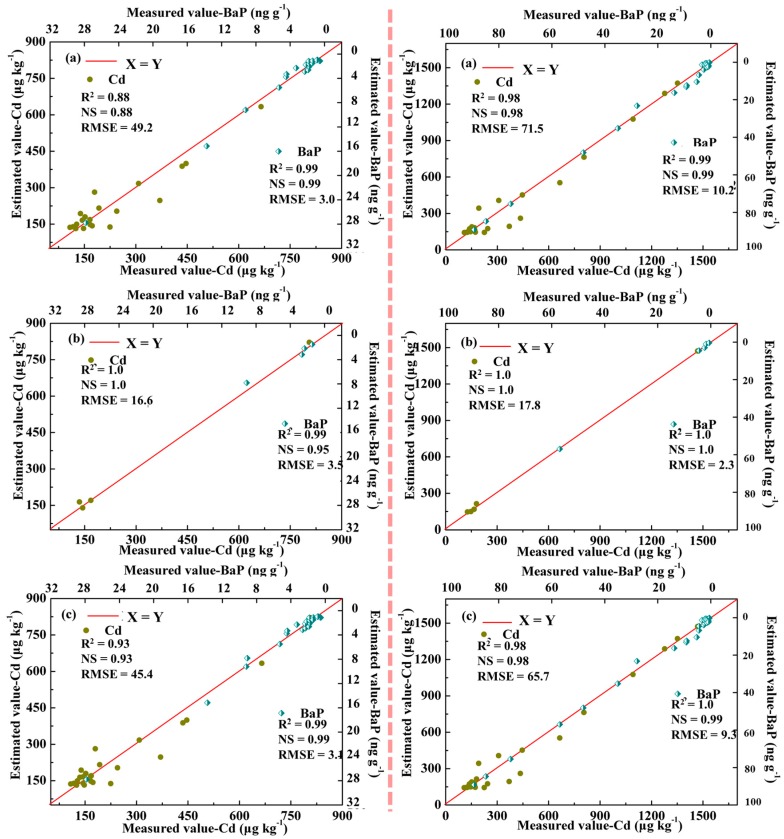
The comparison of the two periods (1950–2003, left; 1950–2015, right) between the measured and estimated value when using the Bayesian regulation back propagation neural network (BRBPNN) model for training (**a**), testing (**b**), and entire database (**c**). NS: Nash-Sutcliffe model efficiency.

During the period 1950–2003, the BRBPNN model estimated the Cd concentration for the database with NS = 0.88 and RMSE = 49.2 for the training samples, NS = 1.0 and RMSE = 16.6 for the testing samples, and NS = 0.93 and RMSE = 45.4 for the total database. The BRBPNN model estimated the BaP concentration for the database with NS = 0.99 and RMSE = 3.0 for the training samples, NS = 0.95 and RMSE = 3.5 for the testing samples, and NS = 0.99 and RMSE = 3.1 for the total database (Figure 4).

### 3.3. Results of the BRBPNN Model

**Figure 5:**
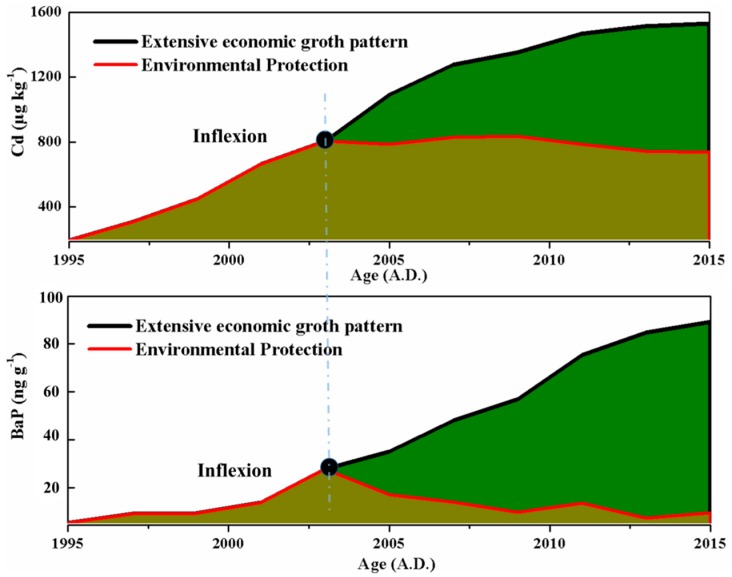
The analysis of the predicted value and the true value of the pollutants during the period 2003–2015. Extensive economic growth pattern relies on an increasing input of production factors to expand production scale and achieve economic growth. In this way, economic growth, higher consumption, higher costs, and low economic returns are achieved. The environmental protection ratio indicates the ratio of the amount of heavy metals removed under environmental protection to the total content.

Under the BRBPNN model, the concentration of pollutants in the sediments of Taihu was obtained under the “extensive economic growth pattern”. As can be observed from the black line shown in Figure 5, the trend of pollutants Cd and BaP is the same; the concentration of pollutants increases year by year, and the rate of the increase is relatively large. In 2015, the concentrations of Cd and BaP reached 1530.6 μg kg^−1^ and 89.2 ng g^−1^, respectively. The red line in Figure 5 shows the actual concentration of pollutants in sediments in Taihu in recent years under various environmental protection policies and measures. The concentrations of Cd and BaP can be seen to increase year by year up until 2003. After 2003, the concentration of Cd remained stable and no longer increased. The BaP concentration not only stopped growing after 2003 but decreased. In 2015, the concentrations of Cd and BaP decreased by 793.2 g kg^−1^ and 79.8 ng g^−1^, respectively, through environmental protection since the 2000s, and the environmental protection ratio of Cd and BaP reached 0.52 and 0.89, respectively.

### 3.4. Prediction

**Figure 6:**
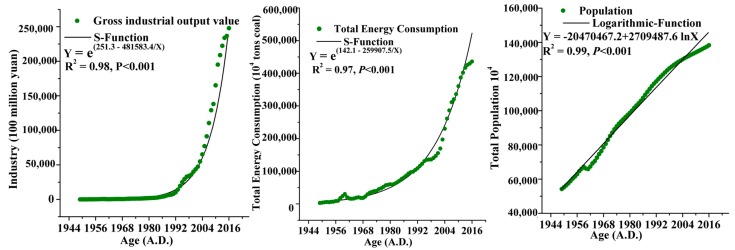
Regression analysis of the gross industrial output value, total population and energy consumption.

The regression analysis of gross industrial output value, energy consumption, and population is shown in Figure 6, and the correlation coefficient R^2^ is 0.98, 0.97, and 0.99, respectively. The significance is *p* < 0.001. Thus, we can obtain projections of the total industrial output value, energy consumption, and population data for the next ten years.

To predict the concentration of pollutants in the sediments of Taihu in the next ten years under the extensive economic growth pattern, the BRBPNN model was established by using the measured values of pollutants during the period 1950–2003 and the predicted values of the pollution during the period 2005–2015. In addition, the BRBPNN model estimated the Cd concentration for the database, with NS = 0.98 and RMSE = 71.5 for the training samples, NS = 1.0 and RMSE = 17.8 for the testing samples, and NS = 0.98 and RMSE = 65.7 for the total database. The BRBPNN model estimated the BaP concentration for the database, with NS = 0.99 and RMSE = 10.2 for the training samples, NS = 1.0 and RMSE = 2.3 for the testing samples, and NS = 0.99 and RMSE = 9.3 for the total database (Figure 4).

The BRBPNN model calculated the concentration changes of pollutants in the sediments of Taihu during the period 2020–2030. As shown in Table 2, under the extensive economic development model, the concentration of BaP is expected to exceed 400 ng g^−1^, and the concentration of contaminant Cd is expected to exceed 2000 μg kg^−1^, which is 20 times that of the natural background value. Environmental protection is becoming more urgently needed. To maintain an environment where there is no serious pollution, greater environmental protection will be required.

## 4. Discussion

Since the economic reform, an extensive economic mode of development has been occurring in China. In the process of promoting rapid economic development, environmental problems have been neglected. The backwardness of science and technology, the waste of raw materials, the direct discharge of exhaust gas, waste liquid and solid waste have led to increasingly serious environmental pollution problems. The historical change in Cd and BaP concentrations in the sediments of Taihu Lake coincided with that before the 2000s. With the promulgation and implementation of a series of environmental laws, China’s environment has changed greatly since the 2000s. The concentrations of Cd and BaP in the sediments of Taihu Lake were controlled (however, although the Cd concentration remained stable, the concentration was nonetheless higher). The BaP concentration showed a downward trend.

**Figure 7:**
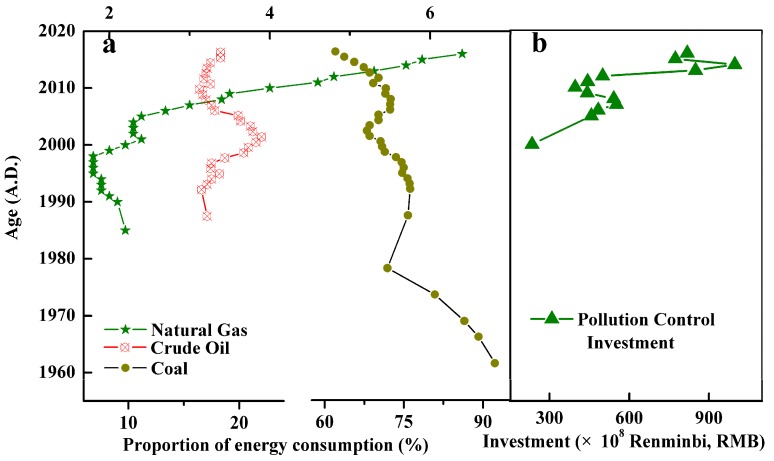
China’s historical energy consumption ratio (**a**); Investment in industrial pollution control in China (**b**).

Many studies [15,16,43,44] have found that the development of industry has indeed brought about increasingly serious environmental pollution, and environmental protection has to some extent curbed the deterioration of the environment. Wan et al. [15] studied the historical changes of heavy metal concentrations in the sediments of lakes in Western China, and he found that under the influence of industrial development since the Reform and Opening-Up in 1978 in China, heavy metals in the ambient atmosphere began to increase in Lake Gonghai. On the whole, their levels were relatively low in the 1980s. In the 1990s owing to accelerated development of the rough and high energy-consuming industries, the atmospheric heavy metals increased the most dramatically. However, in the 21st century the atmospheric heavy metals remained at a relatively high level and this status likely continued or may even have become worse in the following years until the government carried out new stricter management standards for the atmospheric environment. Lei et al. [16,43,44,45] studied the historical changes of pollutant concentrations in the sediments of Eastern Taihu, Changjiang, and Liaodong Bay and considered that 2000 was the turning point of the environmental quality change. This trend is basically consistent with the results of this paper. The possible reasons are as follows: (1) the government’s strong investment; (2) the promulgation and implementation of various environmental protection laws; (3) the enhancement of the people’s awareness of environmental protection; (4) the progress of science and technology, and the utilization of raw materials greatly improved; and (5) the transformation of the type of energy use. As shown in Figure 7a,b, the type of energy utilization in China has been transitioning from primary energy to clean energy, and there has been increasing investment in pollution control. The government’s strong investment [46] in industrial pollution controls reduces the amount of pollutants that directly enter the environment.

In developed countries, such as the United States [47] and Europe [48], industries began very early and rapidly developed, and pollutants also began to increase rapidly during this time period, reaching a peak in the 1970s. With an emphasis on the environment, pollutant accumulations gradually dropped. This trend is similar to that of China, although it occurred nearly 30 years earlier than in China.

With the economy developing rapidly, more raw materials will be put into production, and there will also be more waste gas, waste liquid, and solid waste. The concentrations of Cd and BaP obtained from the BRBPNN model are expected to reach 2015.5 μg kg^−1^ and 407.8 ng g^−1^, respectively. The Chinese government and people will face enormous environmental pressure. This requires the Chinese government and the Chinese people to make greater environmental protection efforts in order to safeguard China’s environment.

## 5. Conclusions

Industry has been developing rapidly and fuel consumption has been increasing rapidly following the reform and opening up of China. Under the extensive economic development model in China, environmental pollution is becoming increasingly serious. The concentrations of Cd and BaP in Taihu Lake reached 805.6 μg kg^−1^ and 27.8 ng g^−1^, respectively, at the end of twentieth century. Correlation analyses and significance tests indicated that Cd and BaP were significantly correlated (*p* < 0.01). For the periods 1950–2003 and 1950–2015, the neural network model estimated the BaP concentration for the database with NS = 0.99 and 0.99 and RMSE = 3.1 and 9.3 for the total database and the Cd concentration for the database with NS = 0.93 and 0.98 and RMSE = 45.4 and 65.7 for the total database, respectively. Through the analysis of the neural network, the concentrations of the pollutants Cd and BaP in the sediments of Taihu Lake have been obtained in the extensive development model (no environmental protection) since the twenty-first century, and the quantitative effect of environmental protection has been obtained. China’s environmental protection measures have achieved remarkable results and have effectively curbed the increase of concentrations of Cd and BaP. However, the concentration of pollutants in the environment is still high, and China’s economy is developing rapidly. Without environmental protection measures, the concentrations of Cd and BaP obtained from the BRBPNN model are expected to reach 2015.5 μg kg^−1^ and 407.8 ng g^−1^, respectively, in 2030. The results from this study suggest that the Chinese government will need to invest more money and energy to clean up the environment.

## Figures and Tables

**Table 1:** China’s related environmental protection laws in the 2000s.

Laws	Time of Promulgation
Law of the People’s Republic of China on the Prevention and Control of Atmospheric Pollution	29 April 2000
Law of the People’s Republic of China on the Prevention and Control of Solid Waste Pollution	29 December 2004
Law of the People’s Republic of China on Grasslands	28 December 2002
People’s Republic of China water law	29 August 2002
Measures for the management of urban green line	9 September 2002
Regulations on the administration of the use of sewage charges	30 January 2002
Regulations on returning farmland to forests	6 December 2002
Clean Production Promotion Law of People’s Republic of China	29 June 2002
Standard for control of hazardous waste storage pollution	28 December 2001
Management method of water function area	30 May 2003
Measures for environmental management of new chemicals	1 April 2003
Interim Measures for administrative licensing of environmental protection	17 June 2004
Supervision and management measures for sewage discharge entrance into river	30 November 2004
Law of the People’s Republic of China on the Prevention and Control of Water Pollution	28 February 2008
Measures for environmental administrative punishment	19 January 2010

**Table 2:** Prediction of pollutant concentrations in sediments of Taihu Lake under the extensive economic growth pattern in the next ten years.

Contaminants	Year										
	2020	2021	2022	2023	2024	2025	2026	2027	2028	2029	2030
Cd (μg kg^−1^)	1631.5	1659.3	1689.7	1722.8	1758.5	1796.9	1837.7	1880.5	1924.9	1970.2	2015.5
BaP (ng g^−1^)	147.7	163.4	180.7	199.9	221.3	244.9	271.1	300.1	332.3	368.1	407.8

## Supplementary Materials

The following are available online at http://www.mdpi.com/1660-4601/15/12/2628/s1, Table S1: Results of recovery and detection limit for BaP and Cd; Table S2: Operating parameters of ICP-MS (ELAN 9000, Perkin-Elmer SCIEX) for the determination of Cd concentrations. Calculation of BRBPNN.
