# Supplementary material for: Influence of Industrialization and Environmental Protection on Environmental Pollution: A Case Study of Taihu Lake, China
Source: Int J Environ Res Public Health. 2018 Nov 23;15(12):2628. doi: 10.3390/ijerph15122628 (PMC6313624; doi:10.3390/ijerph15122628)
Supplement: Supplementary file 1 [file ijerph-15-02628-s001.pdf]

Calculation of BRBPNN

The BRBPNN uses the regularization method to improve its generalization ability. The training objective function *F* is given by the following [1,2]:

$F = \alpha E_W + \beta E_D$

where E_W is the squared sum of the weights in the network, E_D is the squared sum of the residuals between network response values and objective values, and α and β are objective function parameters or hyperparameters.

In the Bayesian framework, the weights of the network are considered to be random variables. At first, the function is set to some prior distribution. When the data have been observed, the posterior distribution of the weights can be updated using Bayes' rule:

$P(w|D, \alpha, \beta, G) = \frac{P(D|w,\beta,G)P(w|\alpha,G)}{P(D|\alpha,\beta,G)}$

where G represents the neural network model, w is the vector of network weights, $P(w|\alpha,G)$ is the prior density, $P(D|w,\beta,G)$ is the likelihood function, and $P(D|\alpha,\beta,G)$ represents the normalization factor [3]. Thus, it can be described as follows:

$Posterior = \frac{Likelihood \cdot Prior}{Evidence}$

Assuming that the weight and data probability distributions are Gaussian, the likelihood function can be expressed as follows:

$P(D|w,\beta,G) = \frac{\exp(-\beta E_D)}{Z_W(\beta)}$

where $Z_D(\beta)$ represents the normalization factor:

$Z_D(\beta) = (\pi/\beta)^{n/2}$

Similarly, the prior probability can be written as follows:

$P(w|\alpha,G) = \frac{\exp(-\alpha E_W)}{Z_W(\alpha)}$

where $Z_W(\alpha)$ is the normalization factor:

$Z_D(\alpha) = (\pi/\alpha)^{k/2}$

Finally, the posterior probability can be written as follows:

$P(w|D, \alpha, \beta, G) = \frac{\exp(-F(w))}{Z_F(\alpha,\beta)}$

We use Bayes' rule to optimize the objective function parameters α and β. Thus, we have the

following:

$P(\alpha, \beta | D,G) = \frac{P(D|\alpha,\beta,G)P(\alpha,\beta|G)}{P(D|G)}$

where P(α, β|G) is the prior probability for the regularization parameters α and β, and P(D|α, β,G) represents the likelihood function, which is called the evidence for α and β [28]. The optimum values for a and b can be inferred, as done by Livingstone (2009) [4]:

$\alpha = \gamma/2E_W$; $\beta = (n - \gamma)/2E_D$; $\gamma = \sum_{i=1}^{m} m - \alpha \cdot trace^{-1}(A)$

where γ is the effective parameter, n is the number of sample sets, m is the total number of parameters in the network, and A is the Hessian matrix of the objective function $F(w)$.

According to Foresee and Hagan [5], the iterative procedure is as follows:

(1) Initialize the values for α, β, and the weights.

(2) Employ one step of the Levenberg–Marquardt algorithm to minimize the objective function $F(w)$.

(3) Compute γ using the Gauss–Newton approximation to the Hessian matrix in the Levenberg–Marquardt training algorithm.

(4) Compute new values for the objective function parameters α and β.

(5) Iterate steps (2–4) until convergence.

References

1. Buiab D T, Lofman O, Revhaug I, et al. Landslide susceptibility assessment in the Hoa Binh province of Vietnam: A comparison of the Levenberg–Marquardt and Bayesian regularized neural networks[J]. Geomorphology, 2012, 171-172(6):12-29.
2. Gao H, Qian X, Zhang R, et al. Bayesian Regularized Back-Propagation Neural Network Model for Chlorophyll-a Prediction: A Case Study in Meiliang Bay, Lake Taihu[J]. Environmental Engineering Science, 2015.
3. Mackay, D. J. C., Bayesian interpolation. *Neural Computation* **1992,** *4*, (3), 415-447.
4. Livingstone, D. J., Artificial Neural Networks: Methods and Applications (Methods in Molecular Biology). *Methods in Molecular Biology* **2008,** *458*, v.
5. Foresee, F. D.; Hagan, M. T. In *Gauss-Newton Approximation to Bayesian Learning*, International Conference on Neural Networks, 1997; 1997; pp 1930 - 1935.

Table S1. Results of recovery and detection limit for BaP and Cd.

Compounds	Average recovery (%)	Detection limit (ng g^{-1})
BaP	104.2	0.414
Cd	106.7	1

Table S2. Operating parameters of ICP-MS (ELAN 9000, Perkin-Elmer SCIEX) for the determination of Cd concentrations.

Parameter	Value	Parameter	value
Nebulizer gas flow (L min^{-1})	0.94	RF power (W)	1100
Analog stage voltage (V)	-1700	Lens voltage (V)	6
Pulse stage voltage (V)	900	Ac rod offset (V)	-6
Discriminator threshold (V)	70	Scan mode	Peak hopping
Speed of peristaltic pump (rpm)	26	Detector	Pulse
Sweeps/Reading	50	Replicates	2
Sampler/Skimmer cones	Nickel	Dwell Time (ms)	2.5
Spray chamber	Ryton® Double-pass Scott-type spray chamber		
Nebulizer	Gem-tip Cross-Flow pneumatic nebulizer		
